# An evaluation of first responders’ intention to refer to post-overdose services following SHIELD training

**DOI:** 10.1186/s12954-024-00957-4

**Published:** 2024-02-13

**Authors:** Saad T. Siddiqui, Anna La Manna, Elizabeth Connors, Ryan Smith, Kyle Vance, Zach Budesa, Jeremiah Goulka, Leo Beletsky, Claire A. Wood, Phillip Marotta, Rachel P. Winograd

**Affiliations:** 1 Missouri Institute of Mental Health, University of Missouri-St. Louis, 1 University Blvd, 206 Benton Hall, St. Louis, MO 63121 USA; 2 SHIELD Training Initiative, Northeastern University, Boston, USA; 3 Department of Social Work, Brown School, Washington University in St. Louis, St. Louis, USA

**Keywords:** Continuity of patient care, Emergency responders, Harm reduction, Opiate overdose, Opioid epidemic

## Abstract

**Background:**

First responders [law enforcement officers (LEO) and Fire/Emergency Medical Services (EMS)] can play a vital prevention role, connecting overdose survivors to treatment and recovery services. This study was conducted to examine the effect of occupational safety and harm reduction training on first responders’ intention to refer overdose survivors to treatment, syringe service, naloxone distribution, social support, and care-coordination services, and whether those intentions differed by first responder profession.

**Methods:**

First responders in Missouri were trained using the Safety and Health Integration in the Enforcement of Laws on Drugs (SHIELD) model. Trainees’ intent to refer (ITR) overdose survivors to prevention and supportive services was assessed pre- and post-training (1–5 scale). A mixed model analysis was conducted to assess change in mean ITR scores between pre- and post-training, and between profession type, while adjusting for random effects between individual trainees and baseline characteristics.

**Results:**

Between December 2020 and January 2023, 742 first responders completed pre- and post-training surveys. SHIELD training was associated with higher first responders’ intentions to refer, with ITR to naloxone distribution (1.83–3.88) and syringe exchange (1.73–3.69) demonstrating the greatest changes, and drug treatment (2.94–3.95) having the least change. There was a significant increase in ITR score from pre- to post-test (*β* = 2.15; 95% CI 1.99, 2.30), and LEO—relative to Fire/EMS—had a higher score at pre-test (0.509; 95% CI 0.367, 0.651) but a lower score at post-test (0.148; 95% CI − 0.004, 0.300).

**Conclusion:**

Training bundling occupational safety with harm reduction content is immediately effective at increasing first responders’ intention to connect overdose survivors to community substance use services. When provided with the rationale and instruction to execute referrals, first responders are amenable, and their positive response highlights the opportunity for growth in increasing referral partnerships and collaborations. Further research is necessary to assess the extent to which ITR translates to referral behavior in the field.

## Background

Fire/Emergency Medical Services (EMS) personnel and Law Enforcement Officers (LEO) are on the front lines of the overdose crisis in the United States. These first responders’ roles offer them unique opportunities to connect with overdose survivors. They can speak with overdose survivors in the moment, provide information about available health and social services, and make referrals to life-saving resources like drug treatment, recovery, and housing services [1, 2]. The connections they can make immediately after an overdose can improve the survival odds of those who have been revived from an overdose in the short term, and provide avenues to treatment and recovery in the long-term [1–3]. First responders who know how to make appropriate referrals can actively intervene with people who use drugs and aid in addressing the ongoing overdose crisis.

First responders who will provide referrals may contribute to interrupting the heightened risk of a subsequent overdose following reversal [4]. Despite the important role LEO and Fire/EMS play in linking survivors to resources through referral, some officers may be reluctant to issue referrals. They may perceive issuing referrals as burdensome, not worthwhile, or not part of their job responsibilities [5–7]. Additionally, stigma among first responders both towards PWUD [8] and towards interventions designed to help PWUD [9] can be a barrier preventing first responders from connecting overdose survivors to timely, post-overdose referrals to care [9]. When missed, these bypassed opportunities may result in survivors missing out on important connections to social services and the health care system [10]. Although it is difficult to influence behaviors directly, it may be possible to increase these first responders’ motivation and intention to conduct referrals during encounters with people who experience overdose [11, 12]. The Safety and Health Integration in the Enforcement of Laws on Drugs (SHIELD) model seeks to address this need by training first responders in public health strategies for working with people who use drugs (PWUD) and demonstrating their value to the responders’ own intrinsic interests in reducing their stress and burnout [13].

Based on the Theory of Planned Behavior (TPB) [13–15], SHIELD trains first responders on public health interventions, overdose response, and post-overdose referrals. The SHIELD model, originally designed for LEO audiences, is based on the idea that when police have the knowledge and resources to provide support to PWUD–and appreciate the benefits of doing so–they will be more willing to act [14, 15]. The training promotes effective public health actions–and reduces the stigma surrounding them–by increasing awareness of the effectiveness of evidence-based, public health-oriented interventions regarding a variety of goals (such as increasing utilization of treatment, reducing injection drug use, and reducing drug-related criminality); awareness of the value to officers’ own intrinsic interests of these interventions; and knowledge of local resources and how to access them. In this study, we use LEO’s intention to refer overdose victims and PWUD to harm reduction and treatment services as a proxy for the number of referrals they intend to make following future overdose reversals [16]. The goal is for LEO to leave SHIELD trainings with a greater willingness and intention to make these referrals [17].

As delivered in Missouri, the SHIELD training consists of three modules taking a total of three hours (both in-person and remotely by Zoom). The first module focuses on first responder resilience and mental health; the second on occupational safety and health concerns related to the overdose crisis (such as risks associated with needlestick injury, bloodborne illness, fentanyl exposure, and overdose rescue); and the third on public health-oriented strategies for referrals (including MAT, peer coaches, harm reduction, and community naloxone distribution) [12, 18]. Following the training, first responders should have the knowledge and ability to make referrals for overdose survivors to local treatment, recovery, naloxone access, and care-coordination services. First responders also learn about the benefits of syringe service programs, and how accessing these services not only reduces risk of injection-related infections such as Hepatitis and HIV, but also increases access to substance use treatment [19, 20].

The SHIELD training was implemented in Missouri by the Connecting the DOTS (Drug Overdose Trust & Safety) project [21]. Although SHIELD was originally designed for LEOs, the curriculum was also adapted for Missouri Fire/EMS audiences. Fire/EMS trainees received a condensed version without law enforcement-specific topics (e.g., encouraging the use of discretion with respect to confiscation of syringes). The curricula for both LEO and Fire/EMS audiences were customized to each training region to help provide first responders with concrete knowledge on locally-available resources and referral pathways. The Fire/EMS training was customized with the same specific, locally tailored referral protocols as the law enforcement training, with the addition of specific EMS-relevant resources.

SHIELD trainings are co-facilitated by an LEO or Fire/EMS trainer peer (depending on the training audience) and a peer specialist with expertise on drug treatment pathways and who has lived experience of opioid use disorder (OUD), overdose events, and recovery. The use of peer trainers has been demonstrated to increase receptiveness to and uptake of training content [22, 23]. SHIELD uses co-facilitation as an educational strategy to (i) model cross-sector collaboration, (ii) provide up-do-date knowledge of the local resource landscape, (iii) humanize addiction, overdose rescue, and the recovery journey, (iv) offer an example of successful recovery, and (v) offer insights on how PWUD may experience interactions with first responders. This co-facilitation strategy, combined with the effort to reduce stigma towards MAT and harm reduction services discussed above, is intended to reduce elements of first responder stigma towards and surrounding PWUD via positive exposure to overdose survivors [24, 25] while avoiding possible pitfalls of direct anti-stigma training analogized from research suggesting that implicit bias trainings can be counterproductive [26].

The present study is a program evaluation of the impact of the SHIELD training on first responders’ intentions to refer overdose survivors to support services. We examined [1] how first responders’ intention to refer was different after the training, and [2] whether first responder profession type was associated with these differences after training. We hypothesized that first responders would endorse intentions to refer overdose survivors more often following the training compared to prior to the training, and that there would be a difference between LEO and Fire/EMS personnel in their intention to refer overdose survivors to substance use-related services.

## Methods

### Procedure

Following Institutional Review Board approval from the University of Missouri-St. Louis, we collected data prior to and immediately following training sessions in Missouri between June 2020 and January 2023. Training sessions were attended by 1731 LEO and Fire/EMS personnel. Inclusion criteria included attending the training, being employed as LEO or Fire/EMS personnel in Missouri, responding to both the pre- and post-training surveys, and being over the age of 18. Participants were excluded if they reported they were recruits currently enrolled in a law enforcement academy or did not provide responses on the outcome of interest. Participants completed online surveys which included questions about demographics, and historical experience with overdose and substance use related calls, and their intention to refer overdose survivors to various substance-use related services.

### Measures

#### Referral history and intention to refer (ITR)

We used five items to assess previous referrals and future intention to make referrals to support services. “Referral” in this context meant provision of a resource with a tangible mechanism for overdose survivors or PWUD to access. On each item, first responders indicated how often they had made (at pre-test) or planned to (at post-test) make referrals to [a] treatment programs, [b] syringe service programs (where available), [c] naloxone distribution programs, [d] social support (e.g., housing, employment) services, and [e] care coordination services (e.g., EPICC program in Missouri [19]). Participants responded using a five-point scale, including [1] “Never”, [2] “Rarely”, [3] Sometimes, [4] Often and [5] “All the time”. Cronbach’s alpha demonstrated good internal reliability for the pre- and post-training scales (*α* = 0.83 and *α* = 0.94, respectively).

#### Professional role and experience

Participants completed training- and agency-specific pre- and post-surveys that indicated whether they were law enforcement or Fire/EMS personnel. At pretest, participants reported personal characteristics including age, gender identity, number of years they have been working in the field, and whether they had personally witnessed an overdose fatality or not. For the item asking about overdose witnessing, participants were able to answer “Yes”, “No”, or "Not Sure”. “Not Sure” responses were treated as “No” for the purposes of the current analysis.

### Statistical analysis

We stratified and compared age, tenure, gender, having ever witnessed an overdose death and pre- and post-test ITR by profession type, using paired t-tests for the continuous variables (age, tenure and ITR) and Chi-square tests for the categorical variables (gender and having ever witnessed an overdose death).

To illustrate pre- and post-test differences for the overall sample and for each profession type, we presented the mean pre- and post-test scores for each ITR item. We did not conduct any statistical tests as a precaution against the likelihood of Type-I errors from multiple pairwise comparisons (between and within time-points, and between and within profession types.

We used a mixed model to examine changes in the intention to refer score between pre- and post-training timepoints and to investigate whether intention to refer varied across types of first responders, while adjusting for random effects between individual trainees and baseline characteristics such as length of tenure and ever witnessing an overdose death [27]. Tenure and ever witnessing an overdose death were included as covariates because of their possible confounding effects on both outcome and predictor variables. Their estimates were not interpreted since they were not the variables of interest and could reflect a multitude of different causal mechanisms operating simultaneously on the outcome [28]. Using intention to refer score as the outcome, we used the following model:

$$\begin{aligned} {Y_{{\text{ITR}}}}\; = \; & {\beta_{0 \cdot ID}}\; + \;{\beta_1}{\text{time}}\,{\text{point}} + \;{\beta_2}{\text{participant}}\_{\text{type}}\; + \;{\beta_3}{\text{witnessed}}\_{\text{od}}\; \\ & + \;\;{\beta_4}{\text{tenure}} + \;{\beta_5}{\text{time}}\,{\text{point}} \cdot {\text{participant}}\_{\text{type}} + \;\left( {{\text{effec}}{{\text{t}}_{ID}}\; + \;\epsilon } \right) \\ {\beta_{0 \cdot ID}}\; = & \;{\beta_0}\; + \;{\text{effec}}{{\text{t}}_{ID}} \\ \end{aligned}$$

We use restricted maximum likelihood (REML) estimation to obtain the model parameters, since this method allows for unbiased estimates of random effect variances [29]. The full model was compared to an intercept-only model with random effects and a fixed effects-only model using AIC and log likelihood to assess goodness of fit. Intercept-only and fixed effect models were refit using maximum likelihood estimation for model comparison [30]. Final model fit was checked using standard linear mixed model fit checks including visual inspection of residuals using Q–Q plots. We completed a post hoc analysis to compare mean ITR across time points and profession types using estimated marginal means and the Tukey method to account for multiple comparisons [31].Our power analysis for an effect size of 0.25 with a power of 80% for multilevel analysis involving two observations per participant required a sample size of 529 participants. All data analysis was conducted using R version 4.3.0 [32], models were fitted using the *lme4* package in R for mixed effects models [30], and goodness of fit statistics and diagnostics (multicollinearity and ICC) were obtained using the *performance* package [32]. Post hoc analysis was conducted using the *emmeans* package [33].

## Results

### Sample characteristics

The project trained 1731 LEO and Fire/EMS personnel, out of which 1223 (70.7%) consented to participate and responded to the pre-training survey. Of these, 932 responded to the post-training survey, representing a retention rate of 76.2%. Based on our inclusion and exclusion criteria, and after matching pre- to post-test responses, we obtained a sample of 742 participants—resulting in a true retention rate of 60.6%—with 1484 total observations (Table 1). Respondents included 524 LEO (71%) and 218 Fire/EMS personnel (29%). The mean age of the sample was 36.75 years (SD = 10.86), and the mean tenure was 11.87 years (SD = 11.04). The sample comprised 614 (84%) men. Participants' mean ITR scores were 2.30 (SD = 0.99) at pre-test and 3.90 (SD = 0.90) at post-test. Pre-training mean intention to refer scores were lower for Fire/EMS participants (*M* = 1.81, SD = 0.72) compared to LEO (*M* = 2.50, SD = 1.01), but post-training mean scores were higher for Fire/EMS (*M* = 3.95, SD = 0.82) than LEO (*M* = 3.88, SD = 0.93) (Table 1).

**Table 1:** Sample characteristics

Characteristic	Overall, *N* = 742^a^	Fire/EMS, *N* = 218^a^	LEO, *N *= 524^a^	*p*-values^b^
Age (Years) [Mean (SD)]	36.75 (10.86)	37.42 (11.87)	36.46 (10.39)	0.500
Tenure [Mean (SD)]	11.87 (11.04)	13.14 (11.78)	11.25 (10.62)	0.300
Gender [n(%)]				0.032
Men	614 (84%)	168 (79%)	446 (86%)	
Women	110 (15%)	42 (20%)	68 (13%)	
Other	6 (0.8%)	3 (1.4%)	3 (0.6%)	
Ever witnessed overdose death [n(%)]	458 (63%)	151 (72%)	307 (60%)	0.002
Pretest ITR score [Mean (SD)]	2.30 (0.99)	1.81 (0.72)	2.50 (1.01)	< 0.001
Posttest ITR score [Mean (SD)]	3.90 (0.90)	3.95 (0.82)	3.88 (0.93)	0.600

^a^Mean (SD); n (%)^b^ Paired t-tests for age, tenure, pre- and post-test ITR scores; Chi-square tests for gender and ever witnessed overdose death

The SHIELD training increased intention to refers scores for both types of participants: for drug treatment programs (2.94–3.95), social support programs (2.75–3.96), care coordination services (2.12–3.98), syringe service programs (1.73–3.69), and naloxone distribution programs (1.83–3.88) and (Table 2). EMS trainees had consistently lower pretest scores than LEO, and consistently higher post-test scores for their intention to refer to each support service.

**Table 2:** Mean Intention to Refer scores for combined sample, Fire/EMS, and LEO (N = 742)

	Combined mean (SD)		Fire/EMS mean (SD)		LEO mean (SD)	
	Pre	Post	Pre	Post	Pre	Post
Drug treatment	2.94 (1.23)	**3.95 (0.96)**	2.34 (1.11)	**4.07 (0.89)**	3.18 (1.20)	**3.90 (0.98)**
Syringe service	1.73 (1.14)	**3.69 (1.12)**	1.33 (0.76)	**3.74 (1.02)**	1.89 (1.22)	**3.67 (1.15)**
Naloxone distribution	1.83 (1.13)	**3.88 (1.05)**	1.53 (0.83)	**3.97 (0.92)**	1.95 (1.21)	**3.83 (1.10)**
Social support	2.75 (1.26)	**3.96 (0.95)**	2.22 (1.04)	**4.03 (0.87)**	2.97 (1.28)	**3.93 (0.99)**
Care coordination	2.12 (1.24)	**3.98 (1.00)**	1.69 (0.89)	**4.14 (0.87)**	2.30 (1.32)	**3.91 (1.04)**

Bold highlighted cells indicate higher values (than the time-point comparison cell); SD = standard deviation

### Intention to refer before and after training

After listwise deletion necessitated by the restricted maximum likelihood method employed in the *lme4* package in R, which requires the measures of interest to be completed in full, the multilevel analysis included 616 participants, with 1184 observations. We use a linear mixed-effects model to examine the pre-training vs. post-training change in 'intention to refer' score. Goodness-of-fit characteristics for the mixed effects model were compared to models with an intercept-only with random effects model, and a fixed effects-only model (Table 3). The mixed effects model had the highest log likelihood ratio, and the lowest AIC value. The adjusted ICC value for the final model was 0.232, compared to 0.207 for the intercept-only model.

**Table 3:** Model comparison

	Intercept model	Fixed effects model	Mixed effects model
*N*	1352 observations 675 groups	1,184 observations	1,184 observations 616 groups
AIC	3636.69	2971.81	2969.55
Log likelihood ratio	− 1809.54	− 1478.91	− 1476.78
Adjusted/conditional *R*^2^	0.56	0.52	0.63
ICC	0.207	–	0.232

The mixed effects model estimates are displayed in Table 4. The influence of profession type on the training effect was statistically significant, with a higher score for LEO versus Fire/EMS (0.509, 95% CI 0.367, 0.651), while the coefficient for time point showed that scores increased from pre-test to post-test (*β* = 2.15; 95% CI 1.99, 2.30). The variance between individuals was 0.17 (± 0.41).

**Table 4:** Final model fixed effect estimates and 95% CI, and random effect variance and SD

Fixed effects	*β*	95% CI	*t* statistic	*p* value
Intercept	1.859	1.708, 2.011	24.057	< 0.001
Within participants (n = 1,184)				
Timepoint	2.151	1.999, 2.302	27.271	< 0.001
Timepoint * Profession type	− 0.669	− 0.854, − 0.484	− 7.091	< 0.001
Between participants(n = 616)				
Profession type (EMS = 0, LEO = 1)	0.509	0.367, 0.651	7.020	< 0.001
Ever witnessed an overdose fatality (No = 0, Yes = 1)	0.043	− 0.074, 0.159	0.716	0.474
Tenure	− 0.006	− 0.011, − 0.001	− 2.486	0.013

Random effects	σ^2^	SD		
Individual	0.166	0.408		
Residual	0.548	0.740		

The asterisk (*) between variables indicates an interaction between the variables

Comparisons of marginal means (Table 5) showed a difference between LEO and Fire/EMS trainees which was significant at both pre-training (*β* = − 0.488; 95% CI − 0.638, − 0.338) and post-training (*β* = 0.181; 95% CI 0.030, 0.332), with LEO having higher pre-training but lower post-training scores than Fire/EMS. Marginal means for Fire/EMS increased from 1.84 (95% CI 1.71–1.96) at pre-training to 3.97 (95% CI 3.84–4.17) at post. For LEO, marginal means increased from 2.32 (95% CI 2.24–2.41) to 3.79 (95% CI 3.70–3.87).

**Table 5:** Post hoc analysis

Timepoint	EMS—LEO contrast estimate (95% CI)	Estimated marginal means (95% CI)	
		EMS	LEO
Pre-training	− 0.509 (− 0.651, − 0.367)	1.81 (1.69–1.93)	2.32 (2.23–2.40)
Post-training	0.148 (− 0.004, 0.300)	3.96 (3.72–3.90)	3.81 (3.72–3.90)

## Discussion

The current study is an evaluation of the implementation of a locally-tailored version of the SHIELD training for law enforcement and an adaptation for Fire/EMS and their effect on these Missouri first responders’ intention to refer overdose survivors to various substance use and social support services. Our findings indicate that completing the SHIELD training is associated with significantly higher intention to refer scores (pretest *M* = 2.30, post-test *M* = 3.90), with higher mean scores for intention to refer overdose survivors and PWUD to drug treatment, syringe service, naloxone distribution, social support (such as housing and employment), and care coordination programs after the training. The results of the mixed effects model suggest that mean overall intention to refer score has an association with profession type (0.509, 95% CI 0.367, 0.651), with LEO reporting greater intentions (than Fire/EMS) to refer prior to the training, but lower intentions immediately after the training. The difference between pre- and post-test was not large for drug treatment referral for either type of first responder, since their pre-training intention to refer score for this type of referral was already high (pretest *M* = 2.94, post-test *M* = 3.95). The largest effects, across both types of trainees, were for referrals for syringe service programs (pretest *M* = 1.73, post-test *M* = 3.69), naloxone distribution (pretest *M* = 1.83, post-test *M* = 3.88) and care coordination (pretest *M* = 2.12, post-test *M* = 3.98).

Participants’ scores suggest first responders are supportive of connecting PWUD to needed services. The high post-test intention to refer to syringe services score for LEOs (pretest *M* = 1.89, post-test *M* = 3.67) was especially interesting since such programs are currently illegal in Missouri [34]. Training content about referral resources and the evidence-base that informs harm reduction may have had an influence on this result. First responders—and LEOs in particular—might accept harm reduction as being in the interest of public health and safety as well as the personal wellness of first responders. Having to visit the same individual for repeat overdose events wears down first responder morale and may lead to burnout [35, 36].

Following the training, Fire/EMS trainees’ intention to refer overdose survivors was significantly improved, especially for care coordination (pretest *M* = 2.22, post-test *M* = 4.03). Fire/EMS trainees had consistently lower pre-training scores than LEO, but higher post-training scores. LEO having higher scores for all intention to refer items at pretest could be attributed to their relationship with Community Behavioral Health Liaisons (CBHL) (previously “Community Mental Health Liaison”) across the state, which predates the DOTS project and provides behavioral health resources for law enforcement responding to overdoses [37]. Since no such collaboration existed for Fire/EMS in Missouri prior to the DOTS project, these personnel may have been unaware of resources and referral mechanisms prior to the training. However, their higher post-test scores suggest they would refer overdose survivors with the clear and specific knowledge of available services they gain from the training. Overall, higher post-training scores for Fire/EMS would suggest that customizations to the original training to include Fire/EMS, as well as the local referral options and instructions provided, were effective aspects of the SHIELD training.

The training hopes to create a custom to refer for first responders, and impart an “implementation intention” whereby the act of referral eventually becomes automated behavior [17]. First responders’ intention to refer is a proxy harm reduction referral behavior based on the Theory of Planned Behavior [15]. The training provides first responders with knowledge and resources to carry out referrals, and addresses stigma towards PWUD, potentially bridging the gap between their intention to refer and actual referral behavior [19, 38].

The DOTS-SHIELD training delivery model described here utilizes three key implementation strategies intended to impact behavioral changes amongst first responders. First, including a peer specialist with lived experience specific to opioid overdose as a trainer, particularly when an emergency first responder reversed their overdose(s), serves to create a bridge between trainees and instructors and humanize the experience of PWUD. Second, providing county-level customization of training content specifically around local resources for substance use treatment, recovery, and harm reduction services serves to create new referral pathways for emergency first responders to connect PWUD to care. Doing this may help to reduce the need for first responders to respond to repeat overdose calls, thus helping to reduce their occupational stress related to PWUD, thus improving their perceptions of overdose survivors. Finally, framing the training through the lens of occupational safety by addressing needle stick injury and fentanyl exposure risk serves to address first responders’ concerns about their own safety when interacting with PWUD. In addition, the SHIELD training has coincided with efforts to increase community buy-in for harm reduction principles and advocacy for policy-level changes to make supportive services more available. These statewide changes, when combined with training on why and how to access new resources, can enable first responders to make effective referrals.

This study was conducted at the same time as increases in acceptance of harm reduction practices throughout the country. City-wide syringe programs have had encouraging results and have garnered buy-in from leadership within agencies patrolling those cities [39, 40]. Additionally, naloxone distribution programs across the country have had success in both lowering opioid overdose deaths and being the fiscally responsible choice [41]. Care coordination efforts, intertwined with first responders overdose reversal efforts, lead to increases in use of support services following an overdose event [42]. This increase in referral attitude runs parallel to the findings that officers believe that referrals are, for the large part, seen as an acceptable practice by their communities and that referrals lead to better future outcomes [19].

### Limitations and future research

Despite the novelty of our program and findings, our study has a number of limitations. Firstly, different trainers conducted training sessions in different settings, which may have affected participants' receptivity and survey responses. The availability and access to local support services (e.g., that ‘underground’ syringe programs only exist in two of Missouri’s 115 counties) may result in varying regional effects of the training, which may also account for the high residual variance in the random effects. Secondly, although the training sessions were mostly conducted in locations with relatively well-developed infrastructures to make most referrals possible, the results may not be generalizable to training programs in more rural settings. Thirdly, even though our response rate was greater than 70%, we had usable data from only 742 out of 1,731 trainees (42.8%), and it is possible that non-response is related to less interest or willingness to work with PWUD. This limitation was further compounded by the reduction of the sample to 616 records with complete information in the final mixed effects model. Fourthly, due to the pre-post design of the evaluation, the results might signify a regression to the mean, which may result in the post-test observed change in scores. Fifthly, the study data was collected over the course of three years (December 2020 to May 2023), during which time there were changes in drug-related policies, public perspectives about opioid overdose and PWUD, and the landscape of the overdose crisis itself, which the current analysis is unable to account for. Finally, without a control group of first responders who did not receive the training, our results provide information about associations between time periods and changes in participants’ intentions to refer, but lack causal evidence. The first responders who attended the SHIELD training may have been amenable towards harm reduction and referral to support services prior to training.

Although a follow-up study on the extent to which intention to refer translates to actual referral behavior by first responders would be required to demonstrate increases in referral to harm reduction and support services, the results of the study are encouraging for advocates of harm reduction principles. A longitudinal follow-up and comparison of trained agencies or their jurisdictions with non-trained agencies would be useful in assessment of the impact of the training on actual referral behavior and treatment uptake. Additionally, the urban or rural status of participating emergency responders would be an important variable in knowledge of referral mechanisms and willingness (and ability) to conduct appropriate service referrals. Such a follow-up could also highlight the most effective aspects and areas of improvement for the training. Finally, the relationships between first responders’ referral intentions and practices, the impact of those referrals on the well-being of PWUD (including repeat overdose events), and the positive and negative ramifications of these outcomes on first responder mental health remain unclear and need further study.

## Conclusion

Successful overdose reversals by first responders will continue to play a critical role in mitigating the opioid overdose crisis, and prevention of repeat overdoses hinges on providing longer term support and care to overdose survivors. Responding to overdoses and being one of the first human contacts for overdose survivors means that Fire/EMS and law enforcement officers are uniquely well-positioned to make a sizable positive difference in this regard. Locally tailored SHIELD training is effective at equipping both types of first responders with the knowledge and resources to carry out referrals to substance use treatment and harm reduction programs. Ensuring that overdose survivors have the option to be connected to treatment and social support resources prevents repeat overdoses, engenders trust of first responders, and reduces overdose deaths. The current study measures the intention of first responders to carry out such referrals, and further research will be necessary to ascertain whether intention translates to actual referral behavior, and, most critically, reductions in repeat overdose events and fatalities.

## Data Availability

The datasets used and/or analyzed during the current study are available from the corresponding author on reasonable request.

## Abbreviations

AIC: Akaike information criterion
DOTS: (“Connecting the DOTS”) Drug overdose trust & safety
EMS: Emergency medical services
ICC: Intraclass correlation
ITR: Intention to refer
LEO: Law enforcement officer
PWUD: People who use drugs
Q–Q: Quantile–quantile
SHIELD: Safety & Health Integration in the Enforcement of Laws on Drugs
TPB: Theory of planned behavior
